# Deep neural network-based automatic metasurface design with a wide frequency range

**DOI:** 10.1038/s41598-021-86588-2

**Published:** 2021-03-29

**Authors:** Fardin Ghorbani, Sina Beyraghi, Javad Shabanpour, Homayoon Oraizi, Hossein Soleimani, Mohammad Soleimani

**Affiliations:** School of Electrical Engineering, Iran University of Science and Technology, Narmak, Tehran 16486-13114 Iran

**Keywords:** Electrical and electronic engineering, Applied physics

## Abstract

Beyond the scope of conventional metasurface, which necessitates plenty of computational resources and time, an inverse design approach using machine learning algorithms promises an effective way for metasurface design. In this paper, benefiting from Deep Neural Network (DNN), an inverse design procedure of a metasurface in an ultra-wide working frequency band is presented in which the output unit cell structure can be directly computed by a specified design target. To reach the highest working frequency for training the DNN, we consider 8 ring-shaped patterns to generate resonant notches at a wide range of working frequencies from 4 to 45 GHz. We propose two network architectures. In one architecture, we restrict the output of the DNN, so the network can only generate the metasurface structure from the input of 8 ring-shaped patterns. This approach drastically reduces the computational time, while keeping the network’s accuracy above 91%. We show that our model based on DNN can satisfactorily generate the output metasurface structure with an average accuracy of over 90% in both network architectures. Determination of the metasurface structure directly without time-consuming optimization procedures, an ultra-wide working frequency, and high average accuracy equip an inspiring platform for engineering projects without the need for complex electromagnetic theory.

## Introduction

Metamaterials, defined as artificial media composed of engineered subwavelength periodic or nonperiodic geometric arrays, have witnessed significant attention due to their exotic properties capable of modifying the permittivity and permeability of materials^1–3^. Today, just two decades after the first implementation of metamaterials by Smith et al.^4^ who unearthed Veselago’s original paper^5^, metamaterials and their 2D counterpart, metasurfaces, have been widely used in practical applications such as, but not limited to, polarization conversion^6,7^, reconfigurable wave manipulation^8,9^, vortex generation^10,11^, and perfect absorption^12,13^. Programmable digital metamaterials remarkably provide a wider range of wave-matter applications which present them especially appealing in the usages of imaging^14^, smart metasurfaces^15,16^, information metamaterials^17–19^, and machine learning applications^20,21^.

However, all of the abovementioned works are based on traditional design approaches, consisting of model designs, trial-and-error method, parameter sweep, and optimization algorithms. Conducting numerical full-wave numerical simulations assisted by optimization algorithm is a time-consuming process that consumes plenty of computing resources. In addition, if the design requirements change, simulations must be repeated afresh, which impedes users from paying attention to their actual needs. Therefore, to fill the existing gaps to find a fast, efficient, and automated design approach, we have taken machine learning into our consideration.

Machine learning and its specific branch, deep learning, are approaches to automatically learn the connection between input data and target data from the examples of past experiences. Machine learning is an effort to employ algorithms to devise a machine to learn and operate without explicitly planning and dictating individual actions. To be more specific, machine learning equips an inspiring platform to deduce the fundamental principles based on previously given data; thus, for another given input, machines can make logical decisions automatically. With the ever-increasing evolution of machine learning and its potential capacity to handle crucial challenges, such as signal processing^22^ and physical science^23^, we are now witnessing their applications to electromagnetic problems. Due to its remarkable potential to provide less computational resources, more accuracy, less design time, and more flexibility, machine learning has been entered in various wave-interaction phenomena, such as Electromagnetic Compatibility (EMC)^24,25^, Antenna Optimization and Design^26,27^, All-Dielectric Metasurface^28^, Optical and photonic structures^29^, and Plasmonic nanostructure^30^.

Recently, T. Cui et al. have proposed a deep learning-based metasurface design method named REACTIVE, which is capable of detecting the inner rules between a unit-cell building and its EM properties with an average accuracy of 76.5%^31^. A machine-learning method to realize anisotropic digital coding metasurfaces has been investigated, whereby 70000 training coding patterns have been applied to train the network^32^. In Ref^33^, a deep convolutional neural network has been studied to encode the programmable metasurface for steered multiple beam generation with an average accuracy of more than 94 %. A metasurface inverse design method using a machine learning approach has been introduced in^34^ to design an output unit cell for specified electromagnetic properties with 81% accuracy in a low-frequency bandwidth of 16-20 GHz. Recently, a double deep Q-learning network (DDQN) to identify the right material type and optimize the design of metasurface holograms has been developed^35^.

**Figure 1:**
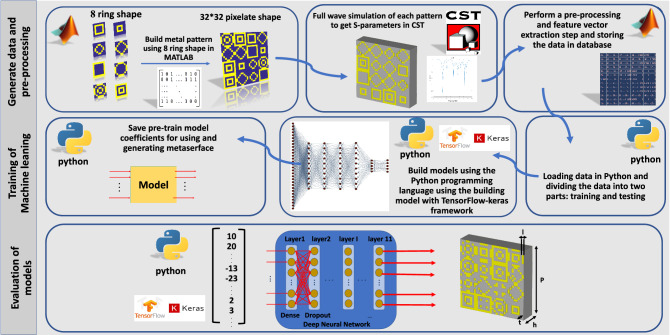
Sketch representation of the design process of DNN-based approach for metasurface inverse design. The process consists of three steps of generating data and pre-processing, training of machine learning, and evaluation of a model.

In this paper, benefiting from Deep Neural Network (DNN), an inverse design procedure of a metasurface with an average accuracy of up to 92 % has been presented. Unlike previous works, to reach the highest working frequency, we consider 8 ring-shaped digital distributions (see top left of Fig. 1) to generate resonant notches in a wide range of working frequencies from 4 to 45 GHz. Therefore, after training the deep learning model by a set of samples, our proposed model can automatically generate the desired metasurface pattern, with four predetermined reflection information (as number of resonances, resonance frequencies, resonance depth, and resonance bandwidths) for ultra-wide working frequency bands. Comparison of the output of numerical simulations with the design target illustrates that our proposed approach is successful in generating corresponding metasurface structures with any desired S-parameter configurations. Determination of the metasurface structures directly without ill-posed optimization procedures, consuming of less computational resources, ultra-wide working frequency bands, and high average accuracy paves the way for our approach to become beneficial for those engineers who are not specialists in the field of electromagnetics; thus, they can focus on their practical necessitates, boosting the speed of the engineering projects.

## Methodologies

### Metasurface design

Figure 1 shows the schematic representation of the proposed metasurface structure consisting of three layers, from top to bottom, as a copper ring-shaped pattern layer, a dielectric layer, and a ground layer to impede the backward transmission of EM energy. FR4 is chosen as the substrate with permittivity of 4.2+0.025i, and thickness of $\hbox {h}=1.5\hbox {mm}$. The top metallic layer comprises 8 ring-shaped patterns distributed side by side, each of which can be divided into $8 \times 8$ lattices labeled as “1” and “0” which denote the areas with and without the copper. Each metasurface is composed of an infinite array of unit-cells. Each unit-cell consists of $4 \times 4$ randomly distributed $8 \times 8$ ring-shaped patterns. Therefore, each unit cell comprises $32 \times 32$ lattices. The length of the lattices, periodicity of unit cells, and thickness of the copper metallic patterns are $\hbox {l} = 0.2$ mm, $\hbox {p} = 6.4$ mm, and $\hbox {t} = 0.018$ mm, respectively. Unlike previous works^31,34^, defining 8 ring-shaped patterns to train the DNN is the novelty employed here to generate the desired resonance notches in a wide frequency band. We designed 8-ring shaped patterns in such a way that the unit-cells generated in the dataset for training the network can generate single or multiple resonances at different frequencies from 4 to 45 GHz, thus, we can import the data set of S-parameters to train the network for our specified targets. It is almost impossible to obtain the relationship between the metasurface matrices and S-parameters. Due to the close connection between the metasurface pattern matrix and its corresponding reflection characteristics, the deep learning algorithm is used to reduce the computational burden for obtaining the optimal solution.

### Deep learning

**Figure 2:**
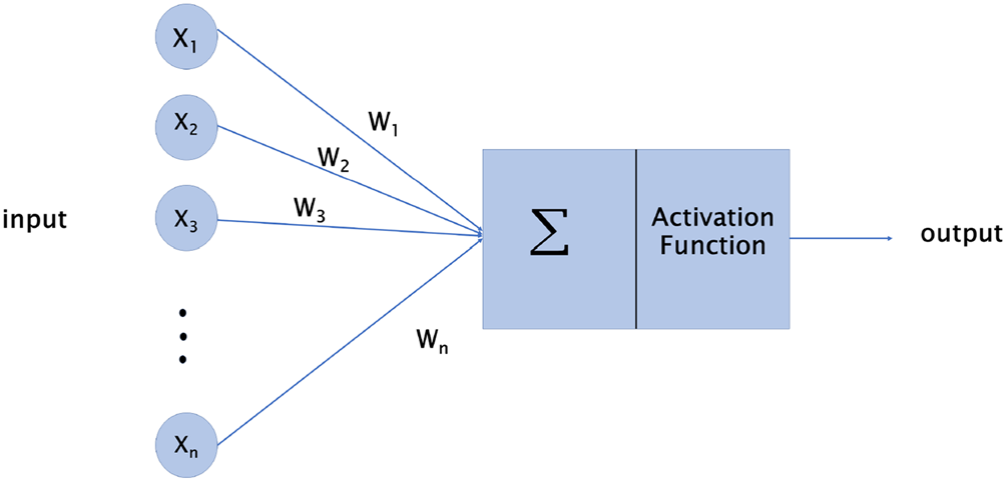
An overview of an artificial neuron.

Artificial neural networks have emerged in the last two decades with many applications, especially in optimization and artificial intelligence. Figure 2 shows an overview of an artificial neuron, with $X_{1}$, $X_{2}$, ... as its inputs (input neurons). In neural networks, each *X* has a weight, denoted by *W*. Observe that each input is connected to a weight; thus, each input must be multiplied by its weight. Then, in the neural network, the sum function (sigma) adds the products of $X_{i}$’s by $W_{i}$’s. Finally, an activation function determines the output of these operations. Then, the output of neurons by the activation function $\phi (u)$, with b as a bias value is:

1 $$\begin{aligned} Y = \phi \left( \sum \limits _{i=1}^n W_{i}X_{i}+b_{i}\right) \end{aligned}$$

The neural network is made up of neurons in different layers. In general, a neural network consists of three layers: input, hidden, and output. A greater the number of layers and neurons in each hidden layer increases the complexity of the model. When the number of hidden layers and the number of neurons increase, our neural network becomes a deep neural network. In this work, we use a DNN to design the desired metasurface.

#### *A. Non-restricted output*

The inverse design of the metasurface is anticipated to determine the intrinsic relationships between the final metasurface structure and its geometrical dimensions by DNN. We have generated 2000 sets of random matrices that represent the metasurface structures using the “RAND” function in MATLAB software. In the next step, we have linked the MATLAB with CST MWS to calculate the S-parameters of the metasurface. To calculate the reflection characteristics of the infinite arrays of the unit cells, we have conducted simulations in which the unit-cell boundary conditions are employed in x and y directions and open boundary conditions in the z-direction. Finally, when it comes to the design procedure, we only need to enter the predetermined EM reflection properties, and our model can generate the output metasurface based on the learned data during the training step. The dataset is established to generate 16 random numbers between 1 and 8 to form $4\times 4$ matrices where each number represents one of the 8 ring-shaped patterns. In the step of “Training of machine learning”, to form our datasets, we have generated two thousand pairs of S-parameter and metasurface pattern matrices (70% as a training set and 30% as a testing set), and the output of the training model is a matrix of $32\times 32$. Each unit-cell can generate 8 notches in the frequency band of 4 to 45 GHz. By defining three features for each resonance (namely, notch frequency, notch depth, and notch bandwidth), the input of our proposed DNN is a vector with dimension 24, and the output is a vector of dimension 1024, which represents a unit cell of $32 \times 32$ pixels. The details of the designed network are summarized in Table 1.

**Table 1:** Detailed information of the non-restricted output network architecture.

Layer number	Layer	Output shape	Number of parameter	Activation function
1	dense_1 (Dense)	(None, 24)	600	Relu
2	dropout_1 (Dropout)	(None, 24)	0	–
3	dense_2 (Dense)	(None, 300)	90300	Relu
4	dropout_2 (Dropout)	(None, 300)	0	–
5	dense_3 (Dense)	(None, 300)	90300	Relu
6	dropout_3 (Dropout)	(None, 300)	0	–
7	dense_4 (Dense)	(None, 300)	90300	Relu
8	dropout_4 (Dropout)	(None, 300)	0	–
9	dense_5 (Dense)	(None, 300)	90300	Relu
10	dropout_5 (Dropout)	(None, 300)	0	–
11	dense_6 (Dense)	(None, 1024)	308224	Sigmoid

In the proposed model, dense and dropout layers are used one after the other (see second step in Fig. 1). In the fully connected (dense) layer, each neuron in the input layer is connected to all the neurons in the previous layers. In the dropout layer, some neurons are accidentally ignored in the training process in order to avoid the misleading of the learning process, as well as increasing the learning speed and reducing the risk of over-fitting. By selecting relevant features from the input data, the performance of the machine learning algorithms is efficiently enhanced. In the proposed model, the values of batch size and learning rate are set to 30 and 0.001, respectively. In addition, the Adam optimization algorithm is used for tuning the weighting values ($W_{i}$). During the training process, the difference between original and generated data is calculated repeatedly by tuning and optimizing the weight values for each layer. When the difference reaches the satisfying predetermined criterion which is defined as loss function, then the training process stops. The Mean Square Error (MSE) is used as a loss function defined as:

2 $$\begin{aligned} {\mathrm{MSE}} = \frac{1}{N}\sum \limits _{i = 1}^N {{{({f_i} - {y_i})}^2}} \end{aligned}$$

where $f_{i}$ and $y_{i}$ denote the anticipated value and the actual value, respectively. Since our desired output in the neural network is 0 or 1, we used the sigmoid function in the last layer, while using other activation functions reduced the accuracy. Formulation of the activation of relu and sigmoid functions are given in Eqs. (3) and (4), respectively:

3 $$\begin{aligned} \phi (x)= & {} {\left\{ \begin{array}{ll} 0 &{} x \le 0 \\ x &{} x > 0 \end{array}\right. } \end{aligned}$$

4 $$\begin{aligned} \phi (x)= & {} \dfrac{1}{1+e^{-x}} \end{aligned}$$

**Figure 3:**
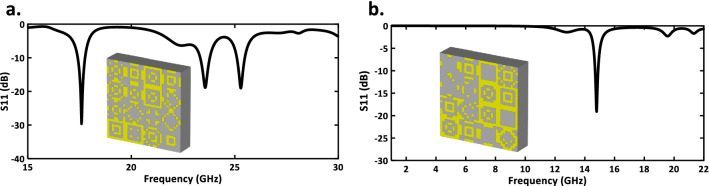
The simulated reflection coefficient of non-restricted output network architecture (**a**) metasurface with three notches under $-10$ dB. (**b**) metasurface with a single notch under $-10$ dB.

In the step of “Evaluation of the model”, for validation, several design goals of S-parameters are suggested, anticipating that our proposed DNN is capable of producing equivalent unit-cell structures. The DNN algorithm is realized by Python version 3.8, and the Tensorflow and Keras framework^36^ are used to establish the model (see the last step in Fig. 1). As an example, a metasurface structure is designed with three notches using the DNN method. The specified reflection information is as follows: [number of resonances; resonance frequencies; resonance depth; and the bandwidth of each resonance] = [ 3; 17.5, 23.5, 25.3 GHz; $-30, -20, -20$ dB; 0.5, 0.5, 0.4 GHz]. Observe in Fig. 3a, that the output full-wave results achieve the design goals.

**Figure 4:**
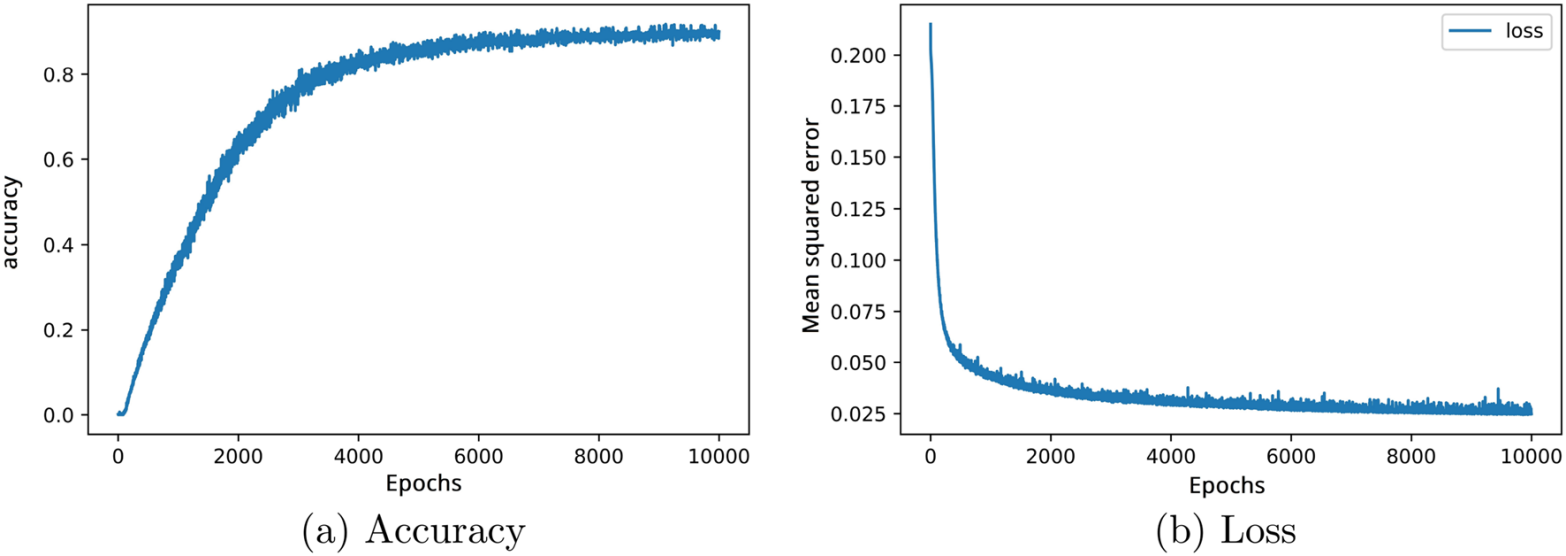
Curves of (**a**) accuracy and, (**b**) loss function relative to 10,000 Epochs for non-restricted network architecture.

For the next example, a uni-cell is designed with one resonance frequency (-15 dB) at 15 GHz. The simulation results show good conformity with our design target (see Fig. 3b). Furthermore, the curves of the mean square error and the accuracy of the presented non-restricted output DNN method are proposed in Fig. 4, where we see that the accuracy rate is higher than 92%.

#### *B. Restricted output*

**Figure 5:**
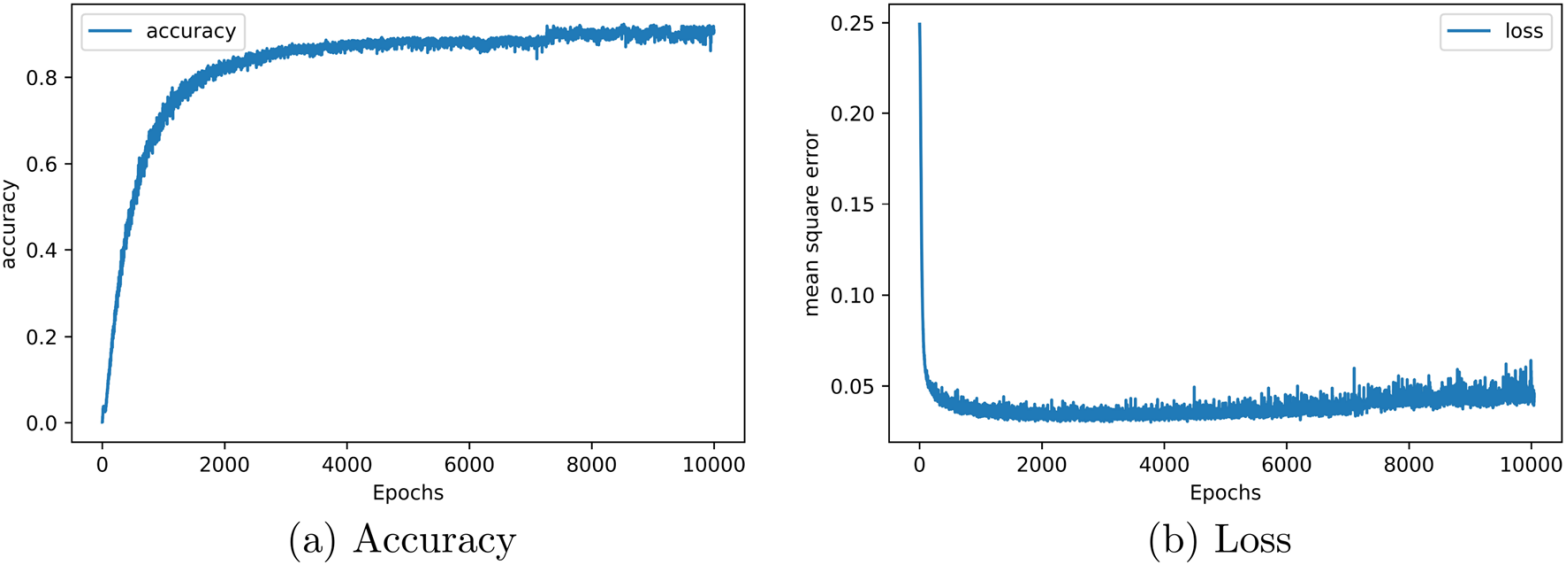
Curves of (**a**) accuracy and, (**b**) loss function relative to 10,000 Epochs for restricted network architecture.

In order to increase the learning speed, reduce the number of calculations, and improve the efficiency of a design process, the network architecture output is restricted in such a way that the DNN should generate the metasurface structure by using the proposed 8 ring-shaped patterns. Unlike the previous approach, in which the output generates a 1024 size vector to form the $32\times 32$ metasurface pixels, in this case the output will generate a 48 size vector. More specifically, each unit-cell consists of $4\times 4$ matrices of these 8 ring-shaped patterns, where each ring-shaped pattern consists of $8\times 8$ pixels. To form the output vector, ring-shaped patterns are denoted by eight digital codes (3-bit) of “000” to “111”. Therefore, the output of the DNN generates a $16\times 3 = 48$ size vector. By restricting the output to produce a 48 size vector, the amount of calculations will be reduced. It will be shown that the accuracy of the network reaches up to 91%. The details of the designed DNN are summarized in Table 2. The other parameters are similar to the non-restricted output network. Figure 5 shows the curves of the loss function and accuracy.

**Table 2:** Detailed information of the restricted output network architecture.

Layer number	Layer	Output shape	Number of parameter	Activation function
1	dense_1 (Dense)	(None, 24)	600	Relu
2	dropout_1 (Dropout)	(None, 24)	0	–
3	dense_2 (Dense)	(None, 500)	12,500	Relu
4	dropout_2 (Dropout)	(None, 500)	0	–
5	dense_3 (Dense)	(None, 500)	250,500	Relu
6	dropout_3 (Dropout)	(None, 500)	0	–
7	dense_4 (Dense)	(None, 500)	250,500	Relu
8	dropout_4 (Dropout)	(None, 500)	0	–
9	dense_5 (Dense)	(None, 500)	250,500	Relu
10	dense_6 (Dense)	(None, 48)	24,048	Sigmoid

**Figure 6:**
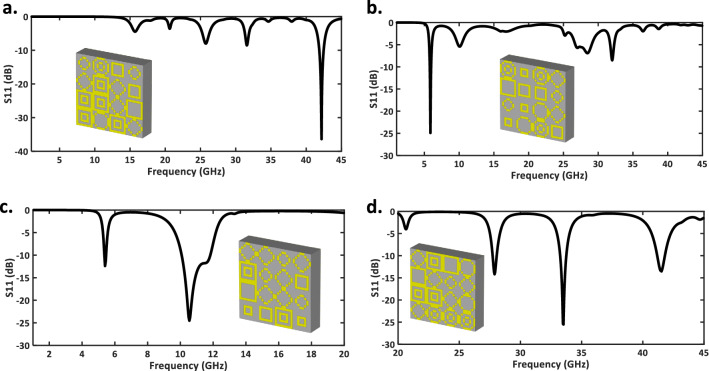
Metasurface design examples through restricted output network architecture.

To further validate the effectiveness of the proposed DNN method for restricted output, four different examples are presented. The specified S-parameters are provided in our network, and the matrix of unit cells are generated through the input S-parameters. We re-enter these matrices into CST MWS to simulate the reflection coefficient of the metasurface. The simulated results are in good accordance with our desired design target (See Table 3 and Fig. 6).

To illustrate the advantages of our DNN approach, as detailed in Table 4, we show the information of training time, time to generate a unit-cell, and the model size for both restricted and non-restricted structures. The results of Table 4 are obtained using Google Colab and with a fixed GPU whose model is Tesla k80 with 13MB of RAM. The design time of our method is about 0.05 sec which is much faster than conventional methods that take about 700 to 800 minutes and even compared to other inverse design methods that used deep learning. Also, our DNN-based approach takes less volume than the conventional method which certifies that our method is more efficient and effective.

Consequently, it has been amply demonstrated that the proposed DNN method is superior to other inverse design algorithms of metasurface structure, from the perspective of computational repetitions, teaching time consumption, and network accuracy. The conformity between the simulated results and design targets promises that the proposed DNN approach is an effective method of metasurface design for a variety of practical applications.

**Table 3:** Desired input targets for four S-parameters, which are presented in Fig. 6.

Examples	Number of notches	Notches frequency (GHz)	Notches depth (dB)	Notches bandwidth (GHz)
Fig. 6a	1	42	$-$35	0.7
Fig. 6b	1	5.8	$-$25	0.2
Fig. 6c	2	5.5, 10.5	$-$12.5, $-$24.5	0.1, 1.8
Fig. 6d	3	28, 33.5, 41.5	$-$14, $-$25, $-$13.5	0.3, 0.5, 0.7

**Table 4:** Information of training time, time to generate a unit-cell, and the model size for both restricted and non-restricted structures.

Training time	Restricted	Non-restricted
	81 min	84 min
Time to generate a unit-cell with pre-trained model	0.052 s	0.055 s
Model size	9 MB	7 MB

## Discussion

Herein, we have proposed an inverse metasurface design method based on a deep neural network, whereby metasurface structures may be computed directly by merely specifying the design targets. After training the deep learning model by a set of samples, our proposed model can automatically generate the metasurface pattern as the output by four specified reflection criteria (namely, number of resonances, resonance frequencies, resonance depths, and resonance bandwidths) as the input in an ultra-wide operating frequency. Comparing the numerical simulations with the desired design target illustrates that our proposed approach successfully generates the required metasurface structures with an accuracy of more than 90%. By using 8 ring-shaped patterns during the training process and restricting the output of the network to generate a 48 size vector, our presented method serves as a fast and effective approach in terms of computational iterations, design time consumption, and network accuracy. The presented DNN-based method can pave the way for new research avenues in automatic metasurface realization and highly complicated wave manipulations.
